# Sensing of Antibiotic–Bacteria Interactions

**DOI:** 10.3390/antibiotics12081340

**Published:** 2023-08-19

**Authors:** Anna A. Baranova, Anton P. Tyurin, Vladimir A. Korshun, Vera A. Alferova

**Affiliations:** Shemyakin-Ovchinnikov Institute of Bioorganic Chemistry, Miklukho-Maklaya 16/10, 117997 Moscow, Russia; anna_ab@ibch.ru (A.A.B.); ap2rin@ibch.ru (A.P.T.); korshun@ibch.ru (V.A.K.)

**Keywords:** antibiotics, sensors, mechanism of action, resistance, phenotypic profiling

## Abstract

Sensing of antibiotic–bacteria interactions is an important area of research that has gained significant attention in recent years. Antibiotic resistance is a major public health concern, and it is essential to develop new strategies for detecting and monitoring bacterial responses to antibiotics in order to maintain effective antibiotic development and antibacterial treatment. This review summarizes recent advances in sensing strategies for antibiotic–bacteria interactions, which are divided into two main parts: studies on the mechanism of action for sensitive bacteria and interrogation of the defense mechanisms for resistant ones. In conclusion, this review provides an overview of the present research landscape concerning antibiotic–bacteria interactions, emphasizing the potential for method adaptation and the integration of machine learning techniques in data analysis, which could potentially lead to a transformative impact on mechanistic studies within the field.

## 1. Introduction

Small molecule antibacterials, also known as antibiotics, originated form the microbial world, where they serve as a weapon of interspecies competition between microorganisms. Antibiotics that act on microorganisms and are low-toxic to macroorganisms are effective therapies for fighting microbial infections. However, antibiotic-resistant pathogenic strains are becoming increasingly common. The spread of antibiotic resistance poses an enormous threat to modern healthcare. To address this problem, both the search for new antibiotics [1,2,3] and rational use of existing antimicrobial agents [4,5] are required. In most cases, antibiotics have multiple mechanisms of action, both primary and secondary, and trigger complex cascades of response reactions in bacteria, which presents a significant challenge for research [6,7].

**Figure 1:**
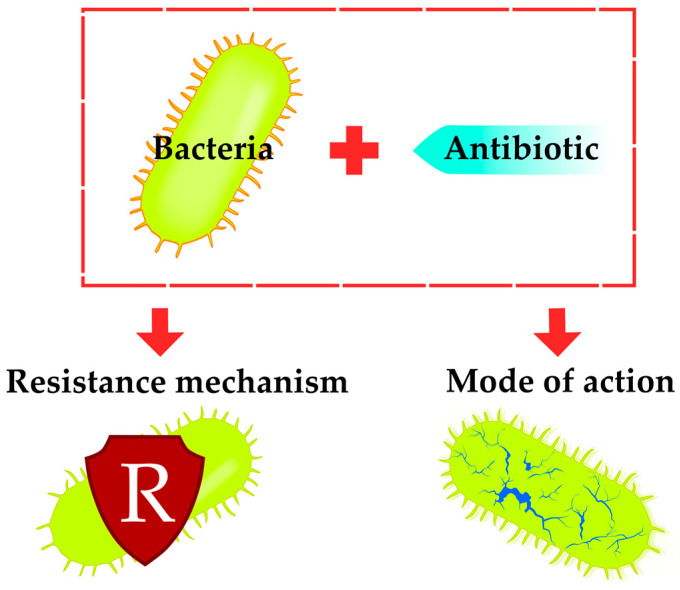
Scope of the review: sensing of antibiotic mechanisms of action (MoA) and mechanisms of resistance.

Modern approaches to addressing the problem of antibiotic resistance require a deep understanding of the fundamental effects of chemotherapeutic agents on the viability of micro- and macroorganisms [1,8,9]. It is through the study of the molecular mechanism of antibiotic action that new drugs can be developed that overcome or do not create resistance [10]. In light of the rapid spread of resistance, there is increasing interest in antimicrobial compounds with multiple targets, and new approaches are needed to establish and validate targets in order to understand such complex mechanisms of action [6]. Fast and reliable sensors for determining the resistance mechanisms of pathogenic microorganisms are necessary for the development of rational personalized therapeutic approaches in antibiotic therapy [11]. This review focuses on the latest advances in sensing and high-throughput methods and approaches to studying the interaction between bacteria and antibacterial agents, mechanisms of resistance and mechanisms of action (Figure 1).

The review covers sensors (chemosensors and biosensors), label-free detection systems and reporter strains for studying the mechanisms of action of antibiotics and resistance to them.

**Figure 2:**
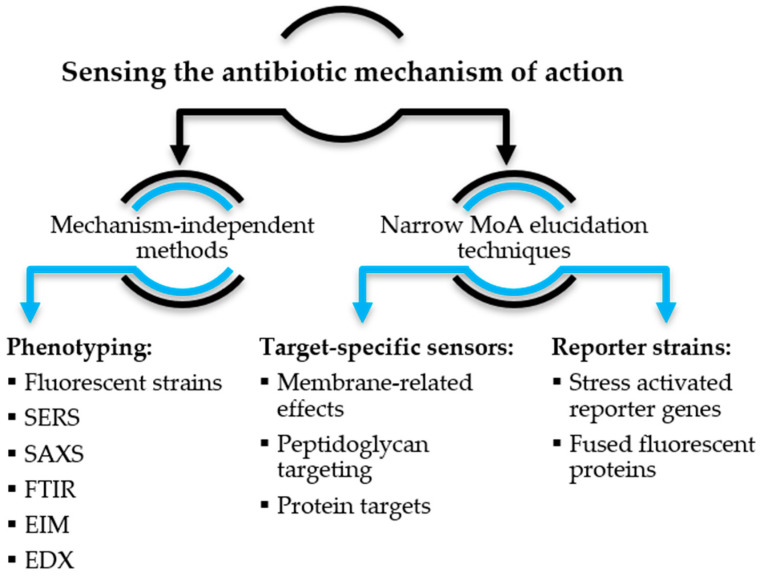
Structure of the review of MoA sensing methods (MoA—mechanisms of action; SERS—surface-enhanced Raman spectroscopy; FTIR—Fourier transform infrared; SAXS—small-angle X-ray scattering; EIM—electro-optical impedance microscopy; EDX—energy-dispersive X-ray).

Omics methods [12] and classical approaches to studying mechanisms of action that do not involve the use of sensor systems are not in the scope of the review. Some aspects of this literature review have been previously covered in specialized reviews, usually from a different perspective. The main focus is on publications from the last five years (2018 to early 2023). Such approaches can be analyzed from two fundamentally different perspectives: technical (by method of signal generation and detection) and biological (by types of studied mechanisms and effects). Since the methods are usually developed to solve specific tasks, for the convenience of readers, the biological approach to classification was chosen, and, within such categories, approaches are divided by the method of signal generation. The first part of the review is devoted to recent developments in MoA studies and is divided into two subsections (Figure 2) on the basis of method specificity.

**Figure 3:**
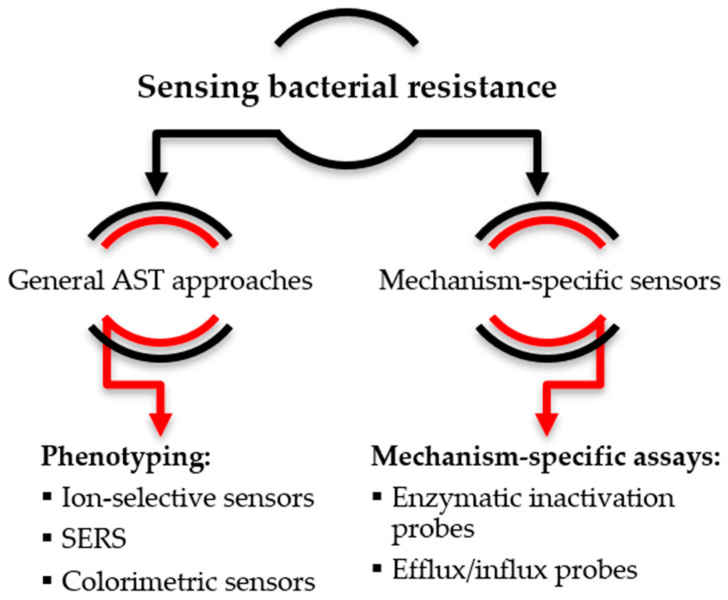
Studies on bacterial resistance summarized in the review (AST—antibiotic susceptibility testing; SERS—surface-enhanced Raman spectroscopy).

The second part of the review is devoted to bacterial resistance. The first section covers non-specific sensors for antimicrobial susceptibility testing, while the second section includes specialized probes for resistance mechanism elucidation (Figure 3).

## 2. Sensing the Antibiotic Mechanism of Action

The mode of action (MoA) refers to the functional outcomes of drug treatment and how the drug attains its intended therapeutic outcomes. For instance, prevailing antimicrobials can be categorized into distinct MoA classes: interference with cell wall function (e.g., penicillins), inhibition of nucleic acid metabolism and repair (e.g., fluoroquinolones), suppression of protein synthesis (e.g., macrolides) and disruption of folate metabolism (e.g., sulfonamides).

Distinct from its MoA, a drug’s mechanism of action is more specific, pinpointing the targets and the precise biochemical interactions (e.g., competitive vs. noncompetitive, agonist vs. antagonist) by which the drug triggers its pharmacological effects. For instance, penicillin’s mechanism of action involves irreversible binding of the β-lactam ring to active sites of penicillin-binding proteins (transpeptidase and acylases), ultimately impeding peptidoglycan cross-linking formation [13].

Target-based approaches are widely used in the search for new antibiotics (both natural and synthetic). It is worth noting that, at present, the introduction of a new drug into clinical practice requires, along with clinical studies, elucidation of its molecular target and mechanism of action. It is not surprising that mechanistic studies attract significant interest in this situation. This section describes the latest achievements in this field.

### 2.1. Mechanism-Independent Methods

**Figure 4:**
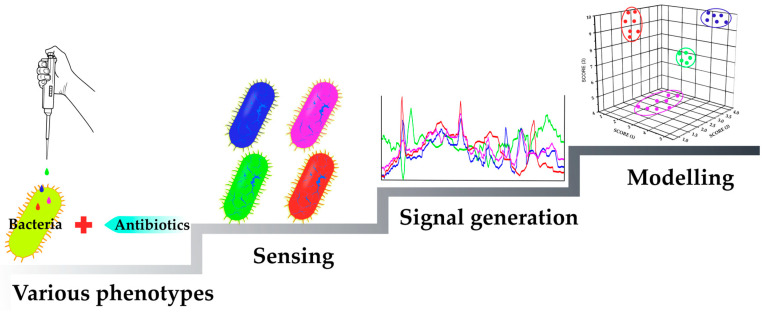
Schematic representation of the mechanism-independent antibiotic screening approach.

The most interesting approach is phenotypic screening without a predefined target-based hypothesis [14]. Morphological profiling is a valuable method for studying the response of both eukaryotic cells and microorganisms to small molecule compounds [15]. Multidimensional profiling has recently attracted increasing attention as a key step in the search for new antibacterial agents [13,16]. This approach involves detecting phenotypic differences in bacteria under the influence of various stressors [17]. In such methods, the studied microorganisms are first subjected to various known types of stress, e.g., antibiotics with well-known mechanisms of action. Analytical signals are obtained from the resulting phenotypes, which are then processed to identify the relationship between the detected parameters and the stress caused to the bacteria. Subsequently, the obtained models allow for typing the organism phenotypes under the influence of the investigated compounds with unknown mechanisms of action (Figure 4).

Recently, such approaches have been actively developed, and systems for phenotypic profiling have been designed based on various signal generators.

#### 2.1.1. Sensing Phenotypes: Fluorescent Stains

**Figure 5:**
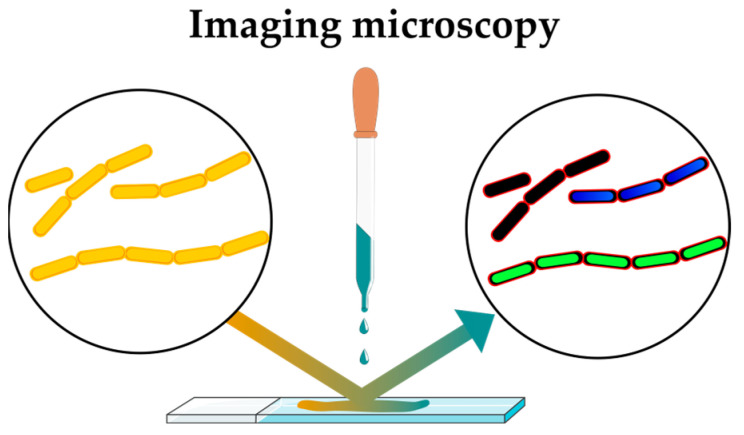
Principle of bacterial cytological profiling (BCP).

One of the most accessible and widespread technologies in signal generation remains fluorescence. There are several approaches based on fluorescent signal detection. First, let us focus on the use of fluorescent stains. Bacterial cytological profiling (BCP) for MoA elucidation of antibacterial agents, introduced 10 years ago [18], still finds diverse applications [19,20,21,22]. In this approach, antibiotic-damaged bacterial cells are stained for DNA, membrane and membrane permeability visualization with fluorescent microscopy (Figure 5). Further image analysis leads to phenotype profiling. Initially, this method was developed for *E. coli*, but it has since been applied to other bacteria, including *Bacillus subtilis* [23], *Staphyloccocus aureus* [24] as well as clinically relevant *Acinetobacter baumannii* strains [25]. Initially, BCP was based on comparing the cytological profiles of the compounds under investigation with those generated under the influence of known antibiotics, which made it unsuitable for studying compounds with an original mechanism of action. Rapid inhibition profiling (RIP) was introduced to overcome this problem [26]. In this method, cytological profiles for unprecedented protein targets are generated via genetic manipulations with the profiled microorganism. This method allows for the identification and characterization of keystone enzymes suitable as antibiotic targets, e.g., in the nucleotide biosynthesis pathway [19].

Recently, cytological profiling has been successfully applied to study the mechanism of rhodanine-containing pan-assay interference compounds (PAINS) [20]. In this study, cytological profiling has established itself as a powerful tool for directing antibiotic discovery efforts. The assay led to the discovery of specific activity against *E. coli* thymidylate kinase among notoriously intractable and nonspecific PAINS analogs. Cytological profiling represents a promising approach for identifying and characterizing novel antibiotics with specific mechanisms of action, ultimately leading to the development of more effective treatments for bacterial infections.

Another remarkable example of phenotyping using this profiling is the study of morphological changes in bacteria under treatment with combinations of antibiotics [27]. It was found that the detected types of morphological changes in bacteria go far beyond simple “synergistic” or “antagonistic” effects, suggesting the possibility of much deeper study of the effects of multiple drugs.

A further direction of development of cytological profiling is the use of time-resolved visualization techniques. Static morphological differences do not fully distinguish the effect of antimicrobials on bacteria. For example, the speed of membrane permeabilization may be crucial for understanding membrane-targeting modes of action [28]. Thus, a quantitative time-lapse fluorescent imaging method called dynamic bacterial morphology imaging (DBMI) was developed [29].

The main focus of the evolution of fluorescent imaging technologies is to improve the quality of images. Therefore, deep learning (DL) approaches were recently widely applied for image processing in bacterial imaging. For example, a system for phenotypic screening at minimal doses using cytological profiling and a machine learning (ML) approach was recently proposed, which allows the identification of weak antibacterial hits [30]. Another ML system was developed [31] and optimized for phenotypic screening [32] which significantly improved the resolution and quality of images and enabled image typing, thus greatly increasing the informativeness of the approach. Further application of AI and ML methods to phenotype sensing data may be a promising direction for future development.

Improving image quality in microscopy continues to increase the capabilities of profiling methods. Specifically, recent single-cell BCP techniques allow the study of heterogeneous populations of bacteria, resulting in the visualization of multiple MoAs with a non-active adjuvant (usnic acid) on the clinically significant bacterium *Acinetobacter baumannii* [33]. It should be noted that, when further expanding the method to study clinical bacterial isolates, heterogeneous cell populations should also be expected. It has been shown that high genetic variation results in the heterogeneous behavior of cells, which can be difficult to study using traditional methods.

The development of fluorescent imaging registration and processing methods is leading to a shift from population-based cytological profiling to visualization of individual cells. This approach enables the study of compounds with multiple mechanisms of action, which are highly promising as agents for overcoming resistance. Recently, a bacterial profiling system with single-cell resolution was developed, which provides an overall accuracy above 90% [34]. The high-content imaging (HCI) technique enables screening multiple cells in high resolution, so it is suitable for the detection of subtle morphological and phenotypic variations. This method was recently applied to phenotyping bacteria under antibiotic exposure. The highest expectations nowadays are related to DL techniques, showing high potential for the development of image-based early drug discovery methods [35].

#### 2.1.2. Sensing Phenotypes: Fluorescent Array Sensors

**Figure 6:**
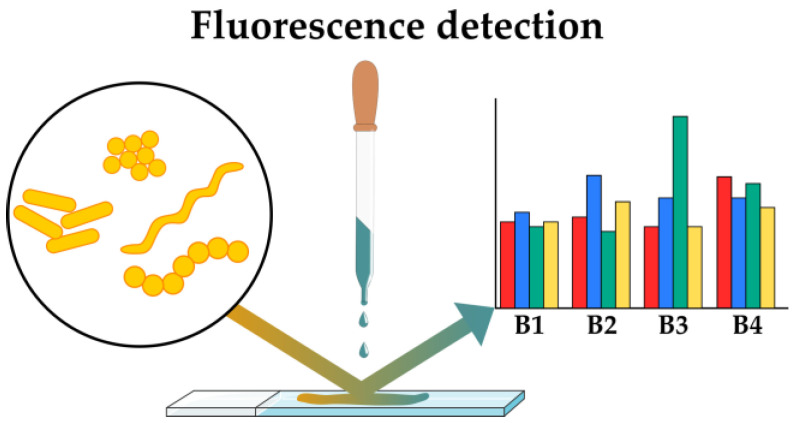
Phenotyping of bacteria under the influence of the studied compounds using fluorescent sensors (B1–B4—various bacteria, colors correspond to various fluorescent signals).

Another approach to phenotypic typing of bacteria under the influence of the studied compounds is the use of fluorescent sensors (Figure 6). These methods utilize fluorescent environment-sensitive probes [36] that are highly sensitive even to subtle changes in the local environment. The dyes exhibit changes in their fluorescence intensities upon the introduction of bacteria, which are caused by changes in local conditions, including pH, polarity, electrostatics and hydrophobicity and supramolecular interactions of the dyes.

Recently, a polymer-based multichannel sensor suitable for high-throughput screening of antibiotic MoA was developed. The sensor includes three solvatochromic fluorophores (pyrene, nitrobenzoxadiazole and REDD) and a cationic benzyl-functionalized recognition element attached to a polymer backbone, poly(oxanorborneneimide). The dye-conjugated polymer was found to be capable of discriminating between different bacteria species. Additionally, the patterns of fluorescent responses on bacteria treated with a set of different antibiotics with established MoAs can be classified into distinct clusters [37].

Another recent example is an ML approach that utilizes a customized array sensor generated by noncovalent conjugation of 2D nanomaterials (fluorescence quenchers) with fluorescently labeled ssDNAs. In the presence of bacteria treated with antibacterials, the fluorescent readout of the sensing elements varies due to the difference in affinity of ssDNA to 2D nanomaterials and bacteria. Analysis of the data with an ML approach has led to an effective mapping of subtle differences in the physico-chemical properties of bacteria [38].

#### 2.1.3. Sensing Phenotypes: Label-Free Methods

Recently, label-free approaches to phenotypic screening based on physico-chemical analysis methods that were previously not used for this purpose have been actively developing. Such development is possible both due to the improvement of the instrumental base and the emergence of more effective data analysis methods.

**Figure 7:**
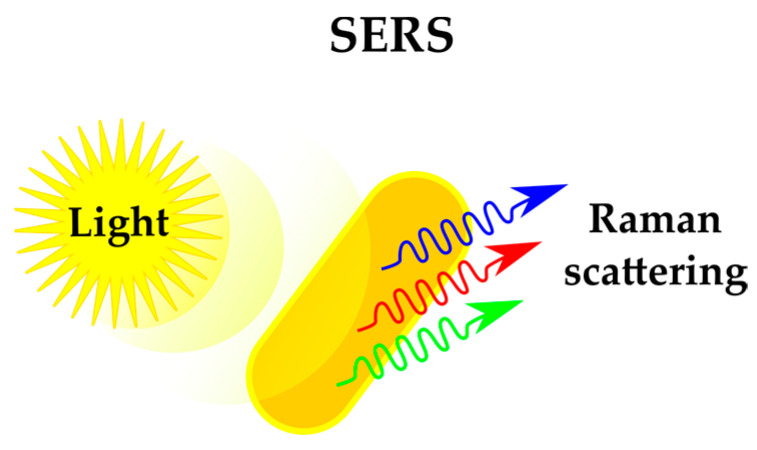
SERS (surface-enhanced Raman spectroscopy) for bacteria phenotyping.

For example, a recent review [39] is dedicated to the use of surface-enhanced Raman spectroscopy (SERS) for profound microbial studies. Raman microspectroscopy is a type of vibrational spectroscopy that exploits the phenomenon of inelastic light scattering. This involves assessing the variance in wavelength between the initial excitation and the emitted light resulting from interaction with the sample, which is governed by molecular vibrations (Figure 7). The resultant wavelength shift offers insights into the molecular bonds within the sample, revealing its chemical composition. [40]. Raman spectroscopy can provide native and rich chemical information on microbial compositions; thus, it is suitable for phenotypic screening. Recent examples of studying chemical stresses for bacteria include the investigation of the effects of biocides on *Aeromonas hydrophila* [41]. SERS was also employed for the evaluation of various inactivation methods on *Pseudomonas syringae* [42].

Despite recent instances of mechanistic studies being rather scarce, SERS-based methods for bacterial phenotyping are rapidly evolving. For instance, bacteria under environmental stresses were successfully differentiated based on SERS signals in a recent study [43]. SERS represents a potentially game-changing technology for our understanding of microbial interactions. Recent progress has allowed the examination of the chemistry of living microorganisms with sub-micrometer precision directly in their natural environment. Furthermore, this method is non-destructive and necessitates no prior sample preparation. [40]. These features also make SERS flexibly compatible with other approaches to provide multidimensional physico-chemical insights into the bacterial world. Therefore, considering previous research [39], SERS remains an attractive technique for further development of mechanistic assays.

**Figure 8:**
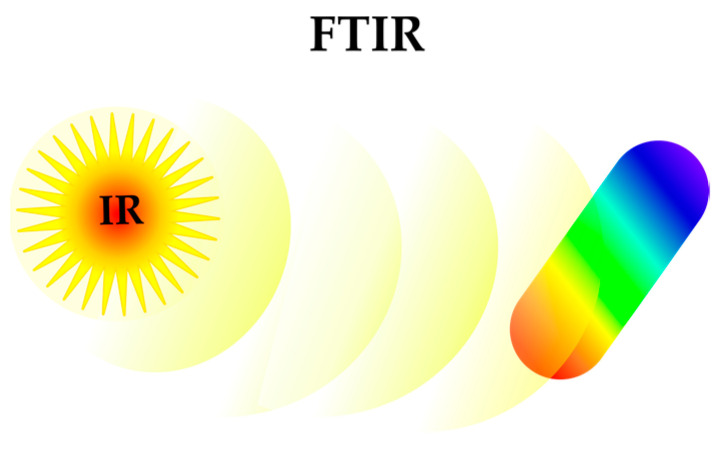
FTIR(Fourier transform infrared) spectroscopy for bacteria phenotyping (IR—infrared).

In the field of organic chemistry, infrared (IR) spectroscopy has established itself as a unique method for generating compound fingerprints by measuring the vibrational energy of the molecular bonds through interaction with infrared radiation. This approach has been successfully applied to obtaining bacterial fingerprints using Fourier transform infrared (FTIR) spectroscopy (Figure 8). The metabolic fingerprints obtained with FTIR spectroscopy were sufficiently specific to provide a clear distinction between the effect of different antibiotics on the *E. coli* metabolism [44]. Further combination of FTIR spectroscopy data with ML-based spectral processing led to increased discrimination accuracy [45]. This approach was further applied to predict MoA and off-target liabilities of antibiotics [46]. FTIR spectra provide molecular insight into most biologically relevant molecules; therefore, FTIR-based methods are a very promising tool in antibiotic development.

**Figure 9:**
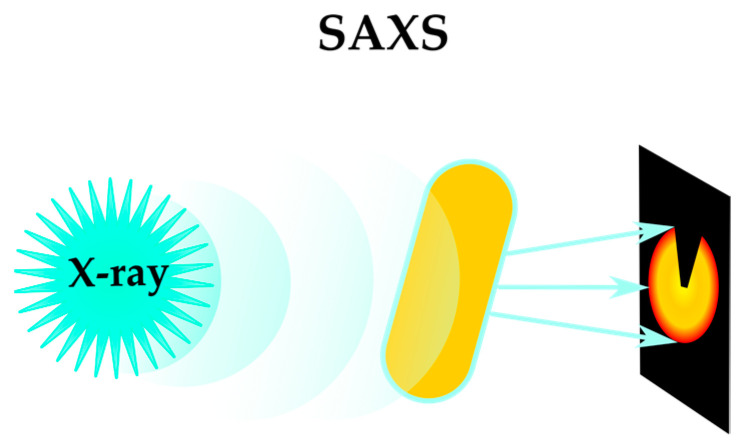
SAXS (small-angle X-ray scattering) for bacteria phenotyping.

Another developing approach to MoA elucidation is small-angle X-ray scattering (SAXS or BioSAXS). Recent developments in instrumentation made SAXS techniques suitable for high-throughput screening. X-ray scattering patterns of *E. coli* treated with different compounds were shown to contain morphological information from the bacteria, illustrating the potential of SAXS to identify the ultrastructural impact of antibiotic treatment on bacterial cells [47,48] (Figure 9). This method was further expanded to compare the differences in protein-induced ultrastructural changes in Gram-negative and Gram-positive bacteria [49]. BioSAXS was successfully applied for mechanistic studies of hybrid peptides [50]. This method is a promising tool in MoA elucidation [51].

**Figure 10:**
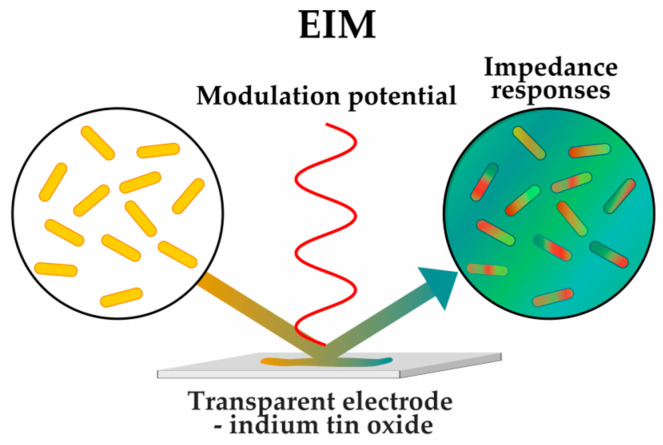
Phenotyping of bacteria using impedance mapping with EIM (electro-optical impedance microscopy, impedance responses are visualized as heat map).

One of the latest approaches to phenotypic screening is the recently described method of imaging single bacterial cells with electro-optical impedance microscopy (EIM) (Figure 10) [52]. This method is based on the dependence of surface optical transmission on local surface charge density. Subcellular impedance mapping reveals the cellular structural changes associated with the antibiotic action [52], making EIM a promising method for further development of MoA elucidation methods.

**Figure 11:**
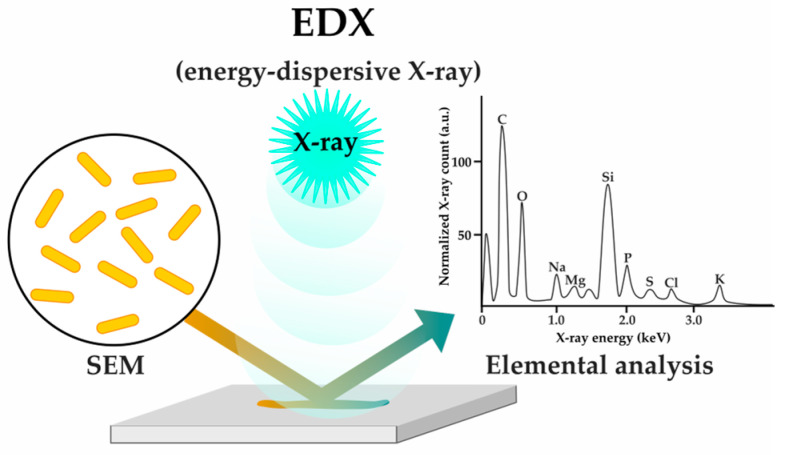
EDX (energy-dispersive X-ray) microanalysis to evaluate bacterial response through elemental analysis monitoring (SEM—scanning electron microscopy).

Another recently applied method for studying bacterial cell responses to various stresses is energy-dispersive X-ray (EDX) microanalysis coupled with scanning electron microscopy (SEM) [53] (Figure 11). This method has been shown to be capable of detecting non-morphological antibiotic effects and is very promising in assessing the early bacterial response [53].

### 2.2. Narrow MoA Elucidation Techniques

#### 2.2.1. Sensing Artificial Phenotypes: Reporter Strains

**Figure 12:**
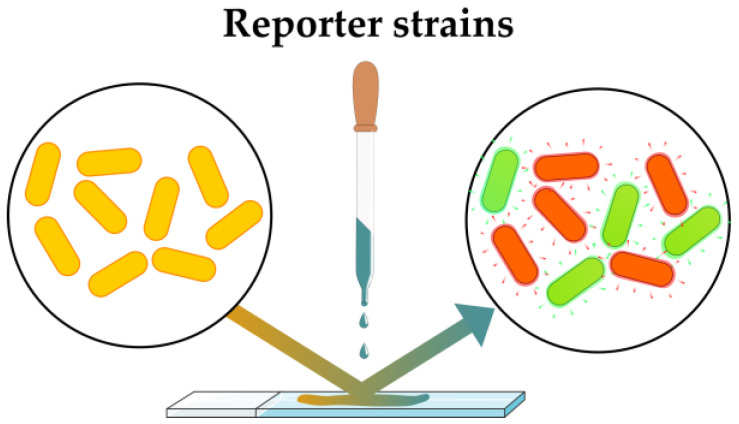
Induction of distinct phenotype changes in reporter strains.

A notable approach involves integrating signal generator biosynthesis genes into the genome of the studied bacterium. Beta-galactosidase (with chromogenic substrate), luciferase and fluorescent proteins are usually used as such signal generators (reporter genes) [54]. Although GFP-tagged bacteria have been used for direct phenotype profiling [55], reporter strains specific to a particular pathway find more diverse applications both for antibiotic screening and for MoA elucidation (Figure 12).

The main advantage of reporter strains for phenotypic screening over the approaches described above is that it is possible to create reporter constructs that will generate a fluorescent signal upon activation of a wide range of stresses in bacterial cells. Phenotypic approaches and other sensing systems are better suited for studying effects related to the bacterial membrane and cell wall. Reporter strains have proven to be effective tools in studying various mechanisms of action, such as protein biosynthesis inhibition or disruption of nucleic acid synthesis [54,56].

Various reporters expressing a fluorescent protein fused with the enzyme of interest are still very informative for mechanistic studies [57,58,59]. This reporter system construction has found wide application for studying *Mycobacterium tuberculosis*, including phenotypic profiling [60,61]. Reporter strains are also widely used for mechanism-informed antibiotic screening [54]. Recent advances in this field include the development of a panel of reporter strains that covers all major MoAs at the initial stage of screening [62].

A fascinating advancement in reporter strain-assisted mechanism-informed screening has been attained through the design of reporter strains tailored for citizen science projects. The pivotal attribute of these strains involves the incorporation of reporter genes that are discernible to the naked eye. This class of reporter genes has the potential to enhance the versatility of the method [63]. 

#### 2.2.2. Sensing of Membrane-Targeting Antibiotics and Other Membrane-Related Effects

Fluorescent stains that localize in the DNA of mammalian and bacterial cells with significantly compromised integrity of the cell membrane are particularly important among the dyes used for visualizing bacterial cells in the phenotypic studies described above. SYTOX green and propidium iodide (PI) are the most used dyes. These sensors continue to be actively used for studying membrane permeabilization [64]. For example, PI has been used to investigate the effects of sub-lethal concentrations of teixobactin [65].

An interesting method was recently used to study the supramolecular assembly of teixobactin. Analogues of teixobactin with Cy3 and Cy5 were used for visualization using fluorescent microscopy. Proximity of two antibiotic molecules at a distance of less than 10 nm and the resulting formation of dimers in bacterial membranes were detected by the presence of Förster/fluorescence resonance energy transfer (FRET) [65].

**Figure 13:**
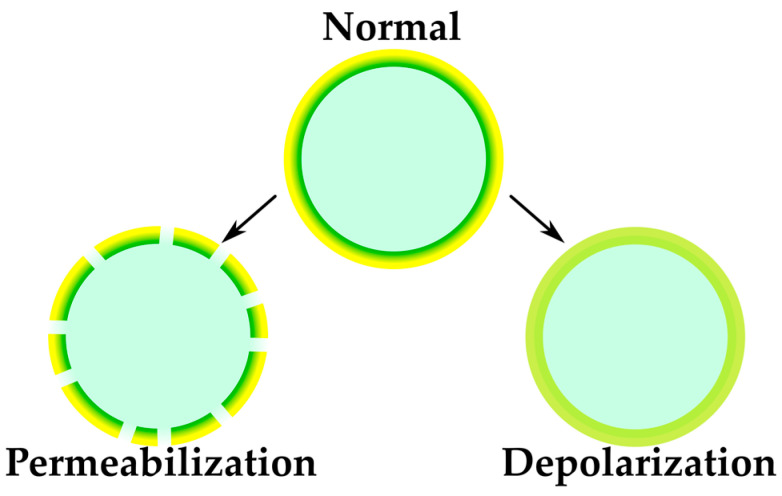
Distinct modes of action of membrane-active compounds.

Compounds that act on bacterial membranes can operate through two separate but interconnected modes: permeabilization and depolarization Permeabilization, as observed with substances like nisin and daptomycin, leads to the creation of pores or disruptions in the membrane structure. Depolarization, on the other hand, is driven by ionophores that transport ions against the concentration gradient defined by the membrane, focusing on the proton motive force (PMF) (Figure 13). To understand the effects of small molecules on bacterial membranes, it is important to distinguish between disruption of membrane potential and membrane permeability. Depolarization is studied using specialized membrane-permeable dyes, such as 3,3′-dipropylthiadicarbocyanine iodide (DiSC3(5)), which have a low fluorescence emission signal when bound to viable bacteria with polarized membranes [64]. Recently, a membrane activity profiling system based on a dual-dye fluorescence assay was developed, using two previously described dyes (TO-PRO-3 iodide and DiOC2(3)) to profile membrane activity in a high-throughput assay [66]. This combination of the dyes was used to study the membrane-associated effects of SCH-79797 [22].

Insertion of particular membrane-disrupting compounds into lipid bilayers can cause dramatic changes in membrane fluidity [67]. To study this effect, there are fluidity-sensitive dyes available, such as laurdan [68]. Recently, this method was used to study a repurposed membrane-targeting antibiotic, bithinol [69].

Biomembranes form the foundation of all cellular compartments, making it crucial to monitor their lipid organization for comprehending cell function and state. Nonetheless, the task of sensing and imaging lipid organization continues to present challenges [70]. Despite these difficulties, successful examples of studying cellular membranes have emerged recently, including sensor systems for detecting phospholipids [71,72], investigating membrane tension [73] and the cellular microenvironment [74,75]. Further development of sensors for exploring the features of bacterial membrane organization can be a promising direction in studying the mechanisms of antibiotic action.

#### 2.2.3. Peptidoglycan Targeting

**Figure 14:**
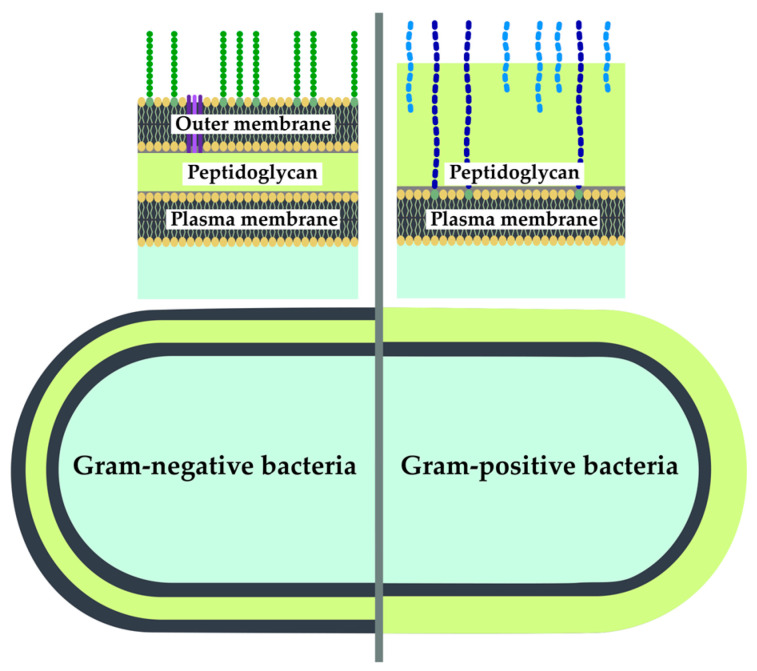
Differences in cell wall organization between Gram-negative and Gram-positive bacteria.

The cell envelope constitutes a multifaceted, multi-layered framework that serves to safeguard and sculpt the cell, impart stability and structural integrity and assumes a pivotal role in facilitating communication with the external environment [76]. In addition, the components of the cell wall are essential for many other processes in the cell. Consequently, targeting bacterial cell wall biosynthesis is one of the important mechanisms of antibiotic action. Moreover, as the cell wall composition differs between Gram-positive and Gram-negative bacteria, it is a suitable target for the development of selective antibacterial agents (Figure 14).

Aside from the previously described fluorescence imaging techniques and phenotypic screening, several approaches to the study of this mechanism of action have been developed recently. Several reporter strains have been developed to monitor stress responses in *E. coli*. A fluorescence-based high-throughput screening assay was used to report σE cell envelope stress and cytosolic heat shock stress as a control and was applied to identify autotransporter biogenesis [77]. A similar assay was also developed to report the activation of Rcs and Cpx responses [78] under cell envelope stress.

Whole-cell biosensors also find diverse applications in recent studies of peptidoglycan targeting. A two-component system responsible for sensing and responding to peptidoglycan targeting in the Gram-negative bacterium *Shewanella oneidensis* was identified. It includes the histidine kinase PghK and the response regulator PghR. On the basis of the PghKR system, a whole-cell biosensor specific to antibiotics that target peptidoglycan biosynthesis was developed [79]. Another recent example is a whole-cell biosensor based on the σ^M^-mediated regulatory system of *Bacillus subtilis* for the detection of antibiotics acting on the cell envelope.

#### 2.2.4. Protein Target Identification

Drug targets are conventionally categorized into six primary classes: enzymes, cell surface receptors, nuclear hormone receptors, ion channels, transporters and nucleic acids. Proteins play a central role in regulating life processes, and, in many instances, natural products primarily target macromolecules like proteins [80]. Consequently, identifying the protein targets of antibiotics holds utmost importance for comprehending the molecular-level mechanism of antibiotic action. Despite the vulnerability of protein-targeting drugs to the development of acquired resistance, there are a lot of conserved essential enzymes attracting interest as targets for antimicrobial agents. For example, membrane sensor histidine kinases contain highly conserved domains and recently were included as a target for the development of inhibitors with broad-spectrum activity and antibiotic adjuvants [81,82,83].

**Figure 15:**
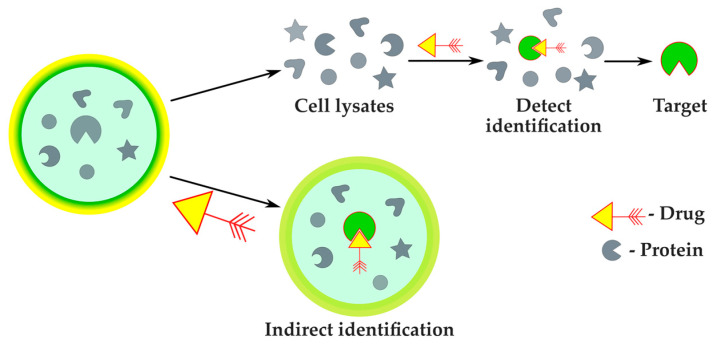
Direct and indirect approaches to protein target identification.

Based on the logical relationships between molecules, targets and phenotypes, the strategies for identifying protein targets are classified into two categories [84]. The first strategy is indirect target identification based on phenotype, covered by previously described methods. The other approach implies direct target identification (Figure 15).

Classical approaches, affinity-based methods (including photo-affinity chromatography) [85,86], various methods of studying unmodified proteins in interaction with small molecules (including those based on protein stability), mass spectrometry [87] and omics approaches [88] still have wide application in the search for and validation of protein targets [84].

However, there are examples of sensor and phenotypic method developments to address this problem. For example, an approach to analyzing phenotypic and mass spectrometric data has been developed. It efficiently identifies antibiotics with a specific target, enabling the discovery of dihydrofolate reductase inhibitors in *Mycobacterium tuberculosis* [89]. Recently, a biosensor based on *Actinomyces oris* was developed to identify sortase inhibitors [90]. Sortase enzymes are attractive targets that attach virulence factors to the surface of bacterial pathogens. *A. oris* exhibits sortase-dependent growth in cell culture and was therefore applied for high-throughput screening of sortase inhibitors.

## 3. Sensing Bacterial Resistance

**Figure 16:**
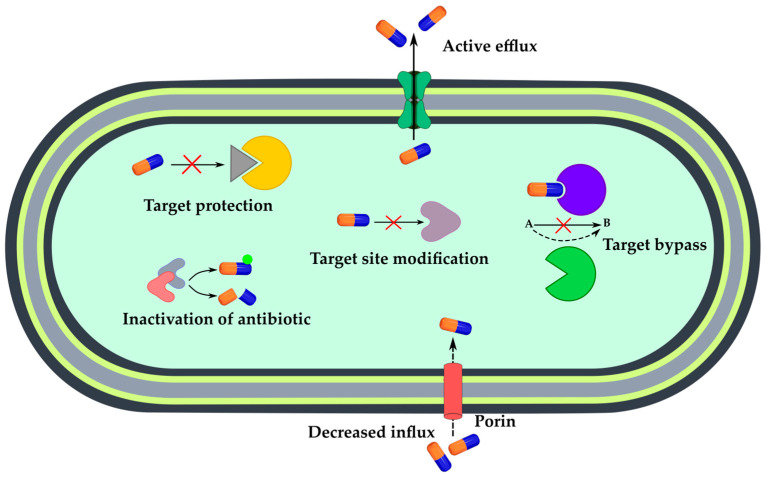
Molecular events involved in the development of antimicrobial resistance.

Another important case of antibiotic–bacteria interaction is the inefficacy of antibiotic action caused by bacterial drug resistance. Molecular events which are involved in the development of antimicrobial resistance are schematically represented in Figure 16 [91]. Enzymatic inactivation or degradation of antibiotics is mediated by chemical modification (hydrolysis, acylation, phosphorylation, adenylation, etc.) of the antibiotic molecule, which prevents binding of the antibiotic to its cell target [92]. Target site alteration is typical for protein targets and can involve mutations in the gene encoding the target protein or post-synthetic enzymatic modification of the binding site. Target bypass is enabled by new protein(s), duplicating the function of the original target but which cannot be affected by the antibiotic, making the antibiotic ineffective due to compensation of antibiotic action. Target protection is a special protection protein interacted with the target protein; the resulting molecular complex cannot be inhibited by the antibiotic [93]. Decreased influx is mediated by changes to membrane, cell envelope or outer glycan layer structure, thus preventing the transport of various compounds, such as antibiotics, into the bacterial cell. For example, this state could be reached by downregulation of porins, which are the main transport proteins. Another way to reduce antibiotic intracellular concentration is an active efflux. This is promoted by transmembrane efflux pumps, which export antibiotics out of bacterial cells [94]. An improved understanding of the origins and spread of drug resistance at the molecular level will facilitate the development of better strategies to manage infections and new effective medicines.

The main determinants of bacterial resistance, like beta-lactam-modifying enzymes (beta-lactamases) and glycopeptide- and polymyxin-related cell wall alterations, have become important for epidemiology over the past decades and are routinely tested for in the clinic [95]. Antibiotic resistance is one of the most urgent problems in modern clinical practice. According to the statistics of the US Centers for Disease Control and Prevention (CDC), resistant strains of microorganisms annually cause at least 2.05 million cases of infectious diseases and at least 23,000 deaths [96].

In the *Global Priority List of Antibiotic-Resistant Bacteria for Research and Development of New Antibiotics* published by the World Health Organization in 2017 [97], the top three rows (“critical priority level”) are occupied by Gram-negative bacteria: carbapenem-resistant *Acinetobacter baumannii*, carbapenem-resistant *Pseudomonas aeruginosa* and resistant to third-generation cephalosporins carbapenem-resistant members of the Enterobacteriaceae family (mainly *Klebsiella pneumoniae*). So far, only polymyxins (colistin, polymyxin B) retain acceptable microbiological activity against many carbapenem-resistant hospital isolates of Gram-negative bacteria. Against the background of a significantly increased consumption of polymyxins for the treatment of infections caused by extremely antibiotic-resistant (extensively drug resistant, XDR) Gram-negative pathogens, an increase in resistance to them is observed [98].

Resistance is a qualitative concept based on a quantitative parameter called minimum inhibitory concentration (MIC). MIC is the lowest concentration of an antibacterial agent that prevents bacterial growth. Classical methods for MIC measurement are broth dilution assay, disk diffusion (Kirby–Bauer) assay and an epsilometer test (E-test). The broth dilution assay involves culturing the test bacterium in a liquid medium with various concentrations of an antimicrobial agent and subsequent growth control. An E-test is a modified Kirby–Bauer method: the main diagnostic criterion of drug susceptibility in these assays is the width of the inhibition zone appearing on the agar medium. The main breakpoints for classification of bacteria as resistant, intermediate or sensitive are based on clinical data and described in CLSI and EUCAST guidelines [99,100,101,102,103,104,105]. The classical approach includes pathogen isolation from the sample, and MIC determination requires a long time (from 24 to 72 h), which is especially critical for slow-growing pathogens like *Mycobacterium tuberculosis*. Saving time and patients’ lives needs more sensitive and fast detection systems, allowing personalized medical care.

### 3.1. Non-Specific Sensors of Bacterial Growth

Non-specific sensing of bacterial growth is now focused on the development of fast, accurate and reproducible methods for antimicrobial susceptibility testing which could be used in medical point-of-care units. The main information obtained from these innovative devices is the same as that from conventional antimicrobial susceptibility testing (AST): phenotypic characterization of bacterial isolates; however, the new-generation methods are much faster. It is a good starting point for studying the mechanism of resistance and rational adjustment of antimicrobial therapy. Non-specific sensors of bacterial replication can be based on different principles: field-effect enzymatic detection [106], glucose metabolization monitoring [107], electrochemical sensing of expressed cytochrome c oxidase [108], plasmonic nanosensors [109], flow cytometry of fluorescent-stained bacterial cells [110,111,112], infrared and Raman spectroscopies [113,114,115,116] and others [117,118,119,120]. Image-based detection systems were developed based on scanning electron microscopy [121], optical microscopy [122], optical video microscopy [122] and other techniques [123]. We further make a brief review the most current trends in AST.

A fully electrical AST method utilizing ion-selective sensors was developed. The sensors detect a pH change in the surrounding medium during the bacterial growth [124]. The electrochemical sensors could be extremely selective and represent a promising alternative to other types of sensors.

Recently, a number of colorimetric assays for phenotyping pathogenic bacteria were developed. Bacteria can utilize D-amino acids (D-AA) in cell wall biosynthesis. This metabolic “omnivorism” was used in the newly constructed colorimetric sensor array for bacterial fingerprinting. Bacterial growth in the presence of D-AA-modified gold nanoparticles as probes triggers the loss of stabilization ligands (D-AA) and aggregation of nanoparticles. The antibiotic-resistant bacteria can be differentiated by analysis the optical response patterns [125]. Another interesting example is a single probe-based dual-mode (colorimetric and photothermal) bacteria fingerprinting approach [126]. This method was applied to differentiate various pathogens, but dual signal detection provides higher accuracy and better differentiation, making it have high potential for AST application.

SERS, previously mentioned as an emerging tool in mechanistic studies, finds diverse application in AST and the detection of bacteria [120,127,128]. This approach has significant advantages, driving significant attention to further development of SERS–AST: label-free, direct detection and live cell imaging, low sample volume, no complex pretreatment, cost-effective techniques with minimal turnaround time. Despite limitations, mainly concerned with low reproducibility and consistency due to the dependence of signal enhancement on multiple factors, SERS-based studies on antibiotic resistance might be a crucial field of future development.

The most conceptually simple but technically difficult approach is miniaturization of broth dilution or agar diffusion assays using microfluidic devices. This emerging technology is still under development, recent results have been reviewed by various groups [129,130,131,132]. Detection of bacterial growth in nanovolumes can be performed much faster than using conventional techniques. The main intriguing advancement of microfluidic sensors of bacterial growth is the ability to work at the level of a single cell [133]. The same trend was previously described for mechanistic studies: recent works tend to study bacterial responses to antibiotic treatment at the level of individual cells. It is envisioned that single-cell analysis techniques could provide more useful data and transform pathogen diagnostics making infection management more effective, fast and personalized [133].

The problem is also relevant for the conventional testing of minimum inhibitory concentration due to the heteroresistance phenomenon [134]. Bacterial heteroresistance is a form of phenotypic heterogeneity when a seemingly susceptible isogenic bacterial population contains resistant sub-populations. The false negative results in AST could be linked to the presence of this heterogeneity type in pathogenic bacterial samples, and it requires additional data to evaluate the effect of bacterial heteroresistance on anti-infective therapy. A recently constructed droplet-based digital MIC screen [134] provides a practical analytical technique for quantifying the single-cell distribution of phenotypic responses to antibiotic treatment.

Another approach for rapid detection of monoclonal and polyclonal bacterial heteroresistance using a motility-sensing microfluidic device was developed [135]. It was found that bacterial motility can be used for direct and rapid identification of bacterial heteroresistance and antibiotic susceptibility. Changes in motility due to the antibiotic treatment happen on a much shorter timescale (<2 h) when compared to bulk bacterial growth (>16 h). The device contained hydrodynamic traps of a particular shape. The platform allows tuning of the trap geometry for a given cell shape. This method allows the detection of heteroresistance in *E. coli* and *Salmonella typhimurium* in 2 h. Moreover, the device can rapidly (1.5 h) quantify the MIC, while simultaneously detecting monoclonal or polyclonal heteroresistance in a bacterial sample [135].

Recently, a microfluidic diagnostic platform for multiplex PCR–allele-specific extension assay for *Helicobacter pylori* heteroresistance was reported. It achieved rapid detection of mutations at positions 2142/2143 in the 23S rRNA gene in a single tube with results readable with the naked eye using a nucleic acid detection strip. This approach allows heteroresistance detection at proportions as low as 0.5%. The platform uses a conventional thermal cycler and is suitable for clinical applications, e.g. for the detection of clarithromycin resistance within 2 h [136].

Quantification of the IE is required for accurate AST [137]. A recent microfluidic screening platform provides the tool to analyze antibiotic susceptibility and the IE at the single-cell level [138]. 

Currently, rapid diagnosis of the resistance profile of an infectious pathogen and a deep understanding of population and single-cell distributions of the resistance levels are urgently needed. Droplet-based microfluidic techniques attract attention as possible solution to this problem [139]. Emerging microfluidic platforms tend to make droplet-based AST suitable for clinical use [132,140,141,142].

### 3.2. Mechanism-Specific Sensors

A deeper understanding of the molecular mechanisms responsible for the observed phenotypic differences is possible only with the help of selective methods. But the arsenal of mechanism-specific methods and probes is very limited, and it is not an area of research and development as dynamic and competitive as fast antimicrobial susceptibility testing.

#### 3.2.1. Enzymatic Inactivation of Antibiotics

**Figure 17:**
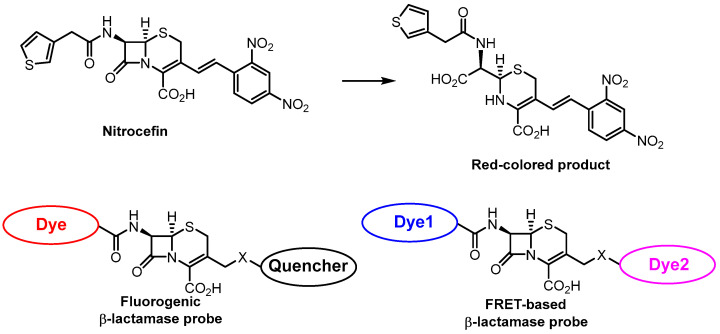
Dye-based β-lactamase probes.

β-Lactam antibiotics are frontline drugs against bacterial infections due to their high efficiency and low toxicity. β-Lactamase, an enzyme synthesized by bacteria resistant to β-lactam antibiotics, can selectively cleave β-lactam rings, which is commonly used for β-lactamase probe construction. The first and most widely used probe for β-lactamase activity testing was nitrocefin (Figure 17), developed in the 1970-s [143]. Cephalosporin-based chromogenic and fluorogenic probes were synthesized and studied as β-lactamase probes. These are usually based on a dye–quencher pair or on a FRET pair of fluorescent dyes (Figure 17). Recent progress in the area is accounted in a review [144]. A recent example includes a bioluminescent system based on luciferin release upon β-lactam hydrolysis [145].

**Figure 18:**
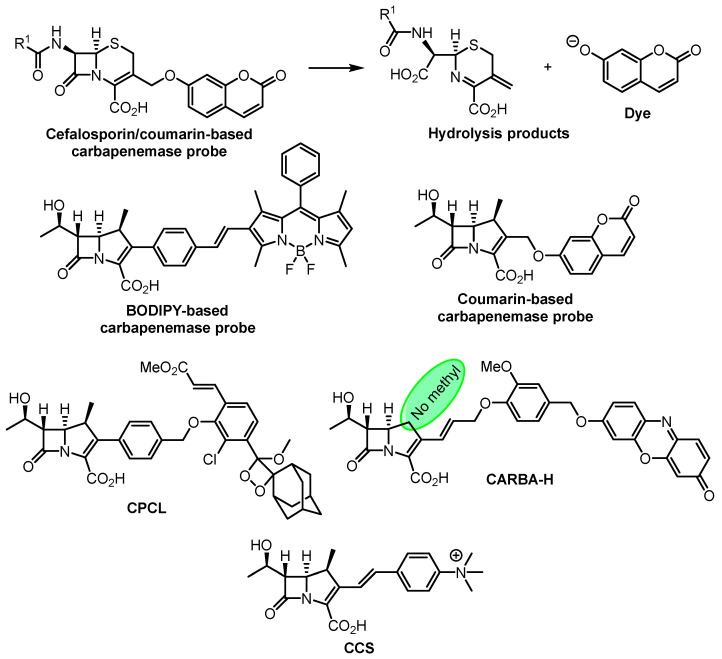
Dye-based β-lactamase probes for carbapenemases.

The development of dye-based probes for carbapenemases has turned out to be rather challenging task. Rao and coworkers reported an original design of fluorogenic probes specific to carbapenemases. It is based on a 6,7-*trans*-cefalosporine skeleton, capable of hydrolysis by carbapenemases, releasing a fluorescent coumarin dye (Figure 18) [146]. The first carbapenem-based fluorogenic probe for carbapenemase detection developed by Xie and coworkers is based on a BODIPY dye (Figure 18) [147]. Fluorescence of the conjugated BODIPY dye is considerably quenched; however, after hydrolysis of the β-lactam ring, the dye part of the molecule undergoes some not fully understood transformations, leading to a >200-fold enhancement of BODIPY fluorescence. Later, the same group developed a coumarin-based carbapenemase probe (Figure 18) [148]. Next, a carbapenemase-sensitive chemiluminescent probe, CPCL (Figure 18), was reported. The ability of the probe to detect a number of clinically relevant carbapenemases, as well as live bacteria with carbapenemase genes, was successfully demonstrated [149]. The fluorogenic probe CARBA-H (Figure 18) allows the detection of a broad spectrum of carbapenemase-producing strains. The absence of the 1β-methyl substituent is essential for carbapenemase activity detection. A clear visual result was obtained within 15 min when the probe was tested against a panel of carbapenemase-producing Enterobacteriaceae strains [150]. The chromogenic probe CCS was recently developed and integrated into a portable paper chip for rapid point-of-care diagnostics [151].

#### 3.2.2. Active Efflux or Decreased Influx

**Figure 19:**
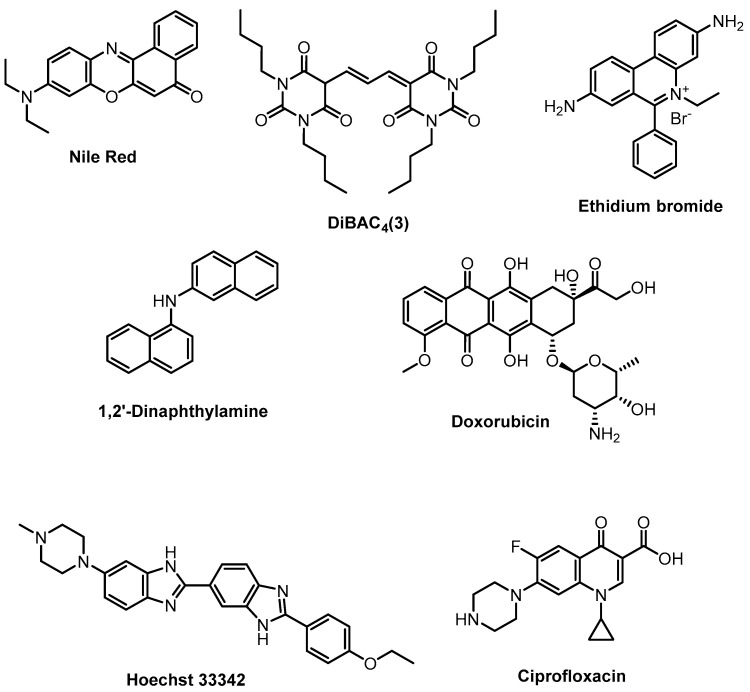
Fluorescent markers for measuring efflux efficacy.

The bacterial cell wall has a dualistic function: on the one hand, it is necessary to prevent the influence of a harsh environment and, on the other hand, to facilitate the access of essential substances. The selectivity of the compounds transported across the cell wall is mediated by a complex system consisting of efflux transporters and specialized uptake mediators [152]. Activation of efflux pumps is an important mechanism of bacterial drug resistance and has been described for many pathogens. Methods for measuring efflux efficacy are usually based on fluorescent markers (Figure 19) and could be split into two groups: for direct measurement of efflux and for measurement intracellular accumulation of a substrate. The main methods were reviewed in an account [153]. Methods for assessing the cellular permeabilization, transport and accumulation of compounds within bacterial cells are continuously evolving and improving [154].

The most commonly used approaches to study antibiotic accumulation in bacteria are based on population-level statistics and cannot be applied at the single-cell level. The heterogenic accumulation could define the population dynamics and other downstream effects of antibiotic pressure. Recently, this problem was addressed with the development of a microfluidic and auto-fluorescence microscopy-based approach to image drug accumulation in individual bacterial cells [155].

The intake of antibiotics through bacterial porine channels is less studied [156]. The main methods used to study the transport, its kinetics and specificity are summarized in the reviews [157,158]. Despite the lack of clarity in understanding these processes, a surprisingly simple model of the Gram-negative bacterial envelope based on starch hydrogel was built to evaluate the penetration of antibiotics into bacterial cells [159]. The model of the membrane is 20% (w/v) potato starch gel, printed on polycarbonate 96-well filter membranes. This model provides rapid permeability testing and may represent a useful surrogate of the Gram-negative bacterial envelope.

## 4. Conclusions and Perspectives

Modern antibacterial drug development is based on an understanding of antibiotics’ molecular mechanism of action and cellular targets. The developed sensing approaches for the identification of these essential features are described in the review. The problem of correct classification of observed antibiotic–bacteria interactions is closely linked with the other, relevant-for-clinical-settings problem of bacterial resistance detection. The first part of this review highlights various upgraded approaches employed for studying the MoA of antibacterial compounds. These methods are summarized in Table 1; the latest advances in the field are emphasizing key trends that could guide future advancements in this field.

The data indicate that this area draws significant attention. In the field of versatile mechanism-independent methods, key trends include (1) expanding the array of physicochemical approaches for phenotyping; (2) improvement of existing methodologies by both instrumental upgrades and advanced data analysis; (3) transitioning towards single-cell profiling. Numerous detection methods have been recently employed in phenotyping, and their continued refinement and integration with established approaches hold the potential to form a foundation for more informative integrated methods, combining multiple techniques. The current advancements in mechanism-specific approaches primarily revolve around the creation of assays with broad applicability.

The second section of the review provides a summary of recent advancements in bacterial resistance studies, which are outlined in Table 2.

As well as in MoA studies, there is a common trend towards shifting modern methods of studying bacterial communities from the population level to single-cell sensing. Recent findings show that, for further development and deepening of the understanding of the interaction between antibiotics and bacteria, it is necessary to study the entire complex of bacterial responses to stress and, therefore, to study all subpopulations of heterogeneous bacterial cultures [8]. Single-cell analysis techniques have the potential to revolutionize pathogen diagnostics and could be applied in personalized medicine and more accurate management of infections in the future [133]. Another significant trend in addressing this issue is the advancement of microfluidic platforms based on various AST approaches. Microfluidics is well-suited for both single-cell monitoring and the accelerated analysis of samples.

The range of phenotyping approaches for AST is notably narrower than that for MoA studies. This can be attributed, in part, to the increased requirement for swift and cost-effective methods in clinical settings. However, continued advancement of these emerging sensing techniques holds the potential to facilitate their integration into medical practices. Successful instances exist where sensory systems designed to distinguish bacterial phenotypic differences have been effectively adapted for use in other fields. For instance, a method initially designed for distinguishing different biofilms using a multi-fluorophore sensor [160] was effectively adapted for bacterial phenotypic profiling [37]. Some strategies for generating distinct signals from diverse phenotypic bacteria are currently being explored for studying the MoA. Bacterial SERS-based techniques have recently shown promise in bacterial detection and species identification indicating their potential for developing novel approaches to investigate antibiotic–bacteria interactions [43,161,162]. Another illustration involves ion-selective silicon nanowire field-effect sensors designed for monitoring pH changes caused by bacterial growth [124]. While this sensor was initially applied for AST, it was also found capable of differentiating bactericidal mechanisms of antibiotics with varying MoAs. Hence, we believe that leveraging methods proven effective in related fields holds significant promise for advancing the understanding of antibiotic–bacteria interactions.

Another prominent trend involves the increasing focus on high-throughput methods, which shifts the bottleneck from data acquisition to data analysis. Furthermore, in the realm of microscopy where image resolution holds a crucial role, the strategies for enhancing it have also notably transitioned from instrumentational upgrades to modern image processing techniques. In the contemporary world, the utilization of AI and ML is progressively gaining importance, encompassing the analysis of scientific data [163]. AI has garnered heightened interest and is being harnessed by chemists to execute diverse tasks in drug discovery [164]. These methodologies have the potential to greatly enhance current techniques for analyzing extensive datasets, capable of handling levels of intricacy that other methods may not address [165,166]. Computational methods for predicting targets are being developed (BANDIT, [167,168]; NeoDTI [169]), including those based on genomic data [170]. Therefore, integration of AI and ML techniques into the study of antibiotic–bacteria interactions can provide new insights and greatly accelerate the development of novel antibiotics. It is important to emphasize that these emerging computational tools require not only extensive datasets for training but also meticulously designed experiments that yield high-quality data. This underscores the need for creating well-curated data repositories and establishing data acquisition standards that can effectively facilitate the advancement of computational methods [13].

## Figures and Tables

**Table 1:** Summary of the methods for MoA studies, discussed in this review, highlighting key trends for further development (MoA—mechanisms of action; SERS—surface-enhanced Raman spectroscopy; FTIR—Fourier transform infrared; SAXS—small-angle X-ray scattering; EIM—electro-optical impedance microscopy; EDX—energy-dispersive X-ray; FRET—Förster/fluorescence resonance energy transfer; MS—mass spectrometry).

Method	Advantages	Disadvantages	Development Points	References
Mechanism-independent approaches				
Fluorescent stains	Informative due to vast variety of dyes	High resolution required demands sophisticated instrumentation	Instrumentation and acquisition upgrades	[29,35]
			Single-cell imaging	[33,34]
	Versatile for various mechanisms		Data processing for enhanced resolution	[30,31,32,35]
Fluorescent array sensors	Versatile for various mechanisms	Construction of array sensors is synthetically complicated	Development of novel array sensors	[37,38]
Label-free phenotyping	Alternative physicochemical methods provide insights in various cellular stress responses	Each method of phenotyping requires specific instrumentation, preventing combination of approaches	Raman scattering (SERS)	[41,42,43]
			Infrared spectroscopy (FTIR)	[44,45,46]
			Small-angle X-ray scattering (SAXS)	[47,48,49,50]
			Impedance microscopy (EIM)	[52]
			X-ray analysis (EDX)	[53]
Mechanism-specific methods				
Reporter strains	Sensitive detection of artificial phenotypic alteration	Narrow spectrum of applicability, each mechanism requires the development of the specific reporter strain	Development of reporter strains with wide applications	[60,61,62]
			Reporter strain for citizen science application	[63]
Membrane-targeting studies	Informative for membrane studies, otherwise difficult to approach	Molecular mode of action can be elucidated only by combination of multiple techniques	FRET-based aggregation probes	[65]
			High-throughput assays	[22,66]
			Novel dye development	[67,68,69,71,72,73,74,75]
Peptidiglycan targeting	Deep insight into cell wall-associated MoA	Narrow spectrum of applicability	Reporter strains	[77,78]
			Biosensors	[79]
Protein target identification	Improved accuracy compared with phenotyping methods	Narrow spectrum of applicability, verification by classic approaches required	MS-assisted phenotyping	[89]
			Biosensors	[90]

**Table 2:** Summary of the methods for resistance studies, discussed in this review, highlighting key trends for further development (SERS—surface-enhanced Raman spectroscopy).

Method	Advantages	Disadvantages	Development Points	References
Phenotyping	Classical approach, providing information on both bacterial growth and susceptibility	Application in medicine requires more rapid and cost-effective methods	Ion-selective sensors	[124]
			Colorimetric and photothermal assays	[125,126]
			SERS	[120,127,128]
			Miniaturization in microfluidic platforms	[133,134,135,136,138,140,141,142]
Inactivating enzymes detection	Sensitive and rapid assay for inactivating enzyme detection	Narrow spectrum of applicability, mostly used for β-lactamase detection	Carbapenemase-selective probes	[146,147,148,149,150,151]
Efflux/influx probes	Well-established method, based on fluorescent probes	Classical approach is suitable only for population-level studies	Single-cell studies	[155]
			Porine modelling	[159]

## Data Availability

Data sharing not applicable.

## Abbreviations

AI: artificial intelligence; AST, antibiotic susceptibility testing; BCP, bacterial cytological profiling; DBMI, dynamic bacterial morphology imaging; CLSI, Clinical & Laboratory Standards Institute; CPCL, Carbapenemase-sensitive chemiluminescent probe; DL, deep learning; DNA, deoxyribonucleic acid; EDX, energy-dispersive X-ray; EIM, electro-optical impedance microscopy; EUCAST, European Committee on Antimicrobial Susceptibility Testing; FRET, Förster/fluorescence resonance energy transfer; FTIR, infrared; GFP, green fluorescent protein; HCI, high-content imaging; IR, Fourier transform infrared; MIC, minimum inhibitory concentration; ML, machine learning; MoA, mode of action; MS, mass-spectrometry; PAINS, pan-assay interference compounds; PI, propidium iodide; PMF, proton motive force; RIP, rapid inhibition profiling; SAXS, small-angle X-ray scattering; SEM, scanning electron microscopy; SERS, surface-enhanced Raman spectroscopy.
